# Appetite Response among Those Susceptible or Resistant to Obesity

**DOI:** 10.1155/2014/512013

**Published:** 2014-03-16

**Authors:** Rachel C. Brown, Rebecca T. McLay-Cooke, Sara L. Richardson, Sheila M. Williams, David R. Grattan, Alexandra W.-A. H. Chisholm

**Affiliations:** ^1^Department of Human Nutrition, University of Otago, P.O. Box 56, Dunedin 9054, New Zealand; ^2^Department of Preventive and Social Medicine, University of Otago, P.O. Box 56, Dunedin 9054, New Zealand; ^3^Department of Anatomy, University of Otago, P.O. Box 56, Dunedin 9054, New Zealand

## Abstract

An alternative approach in determining cause, treatment, and prevention of obesity is to study those who appear resistant to the obesogenic environment. We examined appetite responses in 33 obesity resistant individuals (ORI) versus 28 obesity susceptible individuals (OSI). Fingerprick blood samples to measure ghrelin, total peptide YY (PYY), leptin, glucose, and insulin along with appetite ratings were collected at baseline and 15, 30, 60, 120, and 180 min following consumption of a standardized meal. Fasting, area under the curve (AUC), peak/nadir, and time to peak/nadir were compared. Participants completed the three factor eating questionnaire (TFEQ). No significant differences were observed for ghrelin or PYY. Higher leptin concentrations in the OSI disappeared after controlling for percent body fat (%BF). Significant differences in appetite ratings included a lower hunger nadir among OSI compared with ORI (*P* = 0.017). Dietary restraint (*P* < 0.001) and disinhibition (*P* < 0.001) were lower in ORI compared with OSI, with and without adjustment for %BF. Given the differential body weight of the study groups, similar observed ghrelin concentrations were unexpected, perhaps indicating OSI and ORI respond differently to the same ghrelin concentration. Also ORI response to hunger appears different as they exhibit lower levels of dietary restraint and disinhibition compared with OSI.

## 1. Introduction

Obesity is well-recognised as a disease process leading to multiple pathological consequences. The WHO declared obesity to be an epidemic in 1998 [1]. In countries where data are available, prevalence rates of obesity today are about four times those of thirty years ago [1, 2]. Unfortunately, most strategies to reduce or even curb increases in rates of obesity have been largely unsuccessful [3–6]. Our so-called obesogenic environment has been blamed for the dramatic increase in obesity rates in recent years, as genetics alone cannot account for such dramatic changes in a relatively short time-frame.

Some obesity experts have made dire predictions for future obesity prevalence rates. For instance, Wang et al. [7] have predicted that by the year 2048, all American adults would become overweight or obese. However, evidence suggests that these predictions may be flawed. Information from cross-sectional and prospective studies on temporal changes in body mass index (BMI) indicate that the population distribution of BMI is positively skewed and that over time the degree of skew has increased [8–13]. This means that there is proportionally more shifting at the upper end of the distribution curve with the lower end of the distribution remaining relatively static. That is, within a population, more people are becoming overweight and obese; however, there still remains a substantial sector of the population who have remained lean, seemingly resistant to this obesogenic environment.

Most obesity researchers have investigated the characteristics of overweight and obese individuals and populations in an attempt to determine the cause, treatment, and prevention of obesity. An alternative approach is to study those who have remained lean despite living in an obesogenic environment. Information from this group may allow us to develop novel strategies to benefit those who continually struggle to maintain a healthy body weight.

One potential difference between those who struggle with their weight and those who remain lean with seeming ease may involve appetite regulation. Several hormones have important roles in regulating appetite. For example, ghrelin, an orexigenic peptide, appears important in meal initiation [14], whereas PYY may reduce food intake [15]. A previous study compared concentrations of ghrelin, PYY, glucagon-like peptide 1 (GLP-1), and leptin measured every 4 h over a 24 h period among those considered constitutionally thin (CT) and a group of healthy weight individuals [16]. Individuals were considered CT if they met the following criteria: a BMI between 14.5 and 16.5 kg·m^−2^, stable weight throughout the postpubertal period, presence of menstrual periods without estroprogestative treatment, and the desire to gain weight. Ghrelin, GLP-1, and leptin concentrations were significantly lower, while PYY was significantly higher in the CT group compared to normal weight controls. One could propose that the hormone profile of CT individuals may protect them from overeating and therefore in part explain how thin individuals remain lean despite living in an obesogenic environment. However, it is equally likely that potential differences in eating patterns between the CT and normal weight controls result in changes in the hormone profile.

Some [17–19], but not all [20] research has reported lower baseline and postprandial ghrelin concentrations in obese individuals when compared to their lean counterparts. The suppression of ghrelin in response to a meal may indicate some form of dysregulation of appetite hormones in obese individuals. In addition, ghrelin levels increase with subsequent weight loss, implying a role in long-term weight regulation. It has also been suggested that lower fasting ghrelin may be a consequence of downregulation caused by overeating [21]. Two anorexigenic hormones, Peptide YY and GLP-1, are reported to be lower in obese individuals, suggesting differences in these hormones may be contributing to higher energy intakes in the obese [19, 22, 23].

It would therefore be of interest to compare the hormone profiles of those who report they remain lean with relative ease (ORI) with those who report they struggle to maintain a healthy body weight (OSI). As some studies have shown that ghrelin secretion mirrors reported hunger [14, 20, 24], it would also be worthwhile to investigate any differences in appetite indices between these two diverse groups of individuals.

We investigated hormone concentrations and appetite indices in response to a standard meal among those who remain lean with ease and those who constantly struggle with their weight.

## 2. Materials and Methods

### 2.1. Subjects

Sixty-one participants were recruited from the general public in Dunedin, New Zealand, via the distribution of flyers, designed with specific questions to target obesity susceptible and obesity resistant individuals, around local supermarkets, advertising in the local newspaper, and emails sent to University staff.

To be eligible, participants were required to be healthy males or females aged between 20 and 45 years. Participants completed a screening questionnaire to see if they met our study criteria as either an obesity susceptible individual (OSI) (struggles to maintain their weight, despite perceived low energy intakes) or an obesity resistant individual (ORI) (remains lean with relative ease and can eat whatever they like). Participants were classified as OSI if they answered positively to either or both of the following two statements:

- *I am a person who needs to eat small amounts of food to manage my weight,*
- *I am a person who gains weight easily.*

Conversely, participants were classified as ORI if they answered positively to any of the following statements:

- *I am a person who can eat whatever I like without gaining weight,*
- *I am a person who maintains my weight easily,*
- *I am a person who loses weight easily,*
- *I am a person who finds it difficult to put on weight. *

Participants were excluded if they did not answer positively to any of the screening tool questions, had a thyroid disorder, or were menopausal. In total 28 OSI and 33 ORI were recruited. Obesity resistant individuals had a BMI of 17.5–27.7 kg/m^2^, had always been lean (as indicated by self-reported weight history), and found it difficult to gain but not lose weight. In contrast, OSI had a BMI of 21.6–44.0 kg/m^2^, were likely to experience fluctuations in weight (as indicated by self-reported weight history), and found it difficult to lose but not gain weight. The study protocol was approved by the Human Ethics Committee of the University of Otago, New Zealand. All participants provided written informed consent.

### 2.2. Experimental Design and Procedures

Each participant attended a 4 h clinic visit at the Department of Human Nutrition, University of Otago. Participants arrived at the clinic after an overnight fast of at least 10 h. A fasting fingerprick blood sample using a disposable lancet was taken for measurement of ghrelin (active), PYY (total), leptin, glucose, and insulin. Capillary blood samples were collected in microcentrifuge tubes containing potassium EDTA. This was followed by the consumption of a standardised meal that participants were asked to consume within 15 min. Further capillary blood samples were collected at 15, 30, 60, 120, and 180 min following the start of ingestion of the meal. Participants also completed an appetite questionnaire at each blood sampling time-point.

### 2.3. Standardized Meal

The standardized meal was designed to provide 2440 kJ (584 kcal) for females and 2928 kJ (700 kcal) for males made up of 55, 29, and 15 percent of the total energy intake from carbohydrate, fat, and protein, respectively.The meal was comprised of a muesli cereal (containing oats, wheat germ, Special K (Kelloggs), brown sugar, desiccated coconut, skimmed milk powder, full fat milk, canola oil, almonds, sultanas, dried apricots, sunflower seeds), milk, yoghurt, and orange juice. Participants were required to consume the entire standardized meal. Because the response of important study variables (namely, ghrelin, PYY, and appetite scores) has been shown to be proportional to caloric intake and because this study was cross-sectional, we decided to fix caloric intake for each sex to reduce interindividual variability.

### 2.4. Sampling and Biochemical Analysis

Capillary blood samples (1 mL) were collected in 1.5 mL microcentrifuge tubes containing 10 *μ*L of sodium EDTA. Immediately prior to blood collection, 10 *μ*L of serine protease inhibitor was added for the ghrelin (active) measurement. Upon blood collection the tubes were gently inverted and stored on ice. Samples were then centrifuged for 15 min to obtain plasma, which was stored in microcentrifuge tubes at −80°C until assay. Whole capillary blood was also collected into a HemoCue cuvette and blood glucose concentration measured using a HemoCue Glucose 201+ Analyzer (Helsingborg, Sweden).

Ghrelin (active), PYY (total), leptin, and insulin were analysed using immunoassay (Human Gut Hormone Panel LINCOplex Kit, LINCO Research, St. Charles, MO, USA). The minimum detectable concentrations for the hormones were 1.8 pg·mL^−1^ for ghrelin (active), 8.4 pg·mL^−1^ for PYY (total), 157.2 pg·mL^−1^ for leptin, and 44.5 pg·mL^−1^ for insulin. The coefficient of variation for measurements of ghrelin, PYY (total), leptin, and insulin was 13.0%, 8.1%, 11.8%, and 8.4% respectively.

Area under the curve (AUC) for ghrelin, PYY (total), leptin, glucose, and insulin was calculated by the trapezoid method.

### 2.5. Appetite Ratings

At each blood sampling time-point participants completed a series of appetite related questions using a 10 cm visual analogue scale (VAS) [25, 26]. In relation to each question, there were extreme states anchored at either end of the line. The questions asked were “how hungry are you right now?” (not at all hungry/as hungry as I've ever felt); “preoccupation with thoughts of food” (no thoughts of food/very preoccupied, difficult to concentrate); “how strong is your desire to eat right now” (very weak/very strong); and “how full are you right now?” (not at all full/as full as I have ever felt). The VAS was measured by an investigator blinded to the study group. Area under the curve (AUC) for each rating was calculated by the trapezoid method. The observed peak/nadir and time to peak/nadir were recorded. The three-factor eating questionnaire (TFEQ) [27] was administered on a separate occasion before the 4 h clinic visit.

### 2.6. Body Composition

Body weight was measured on calibrated electronic scales (Wedderburn) that measured to the nearest 0.01 kg. Height was measured to the nearest millimeter using a stadiometer. Body composition including lean mass, fat mass, and body fat percentage (%BF) was measured using dual-energy X-ray absorptiometry (DXA) (manufacturer info DPX-L Scanner, Lunar Corp., Cincinnati, OH, USA) using software version 1.35 (Lunar, Cincinnati, OH, USA) at the Dunedin Public Hospital Dual X-Ray Absorptiometry Scanning Unit. Following screening, participants also completed a questionnaire regarding past-weight history. Weight history information was not used to further categorise the participants, but it did confirm their status as obesity resistant or obesity susceptible. When entering the study participants self-reported being weight stable.

### 2.7. Statistical Analysis

The primary outcome measure to be assessed was the postprandial change in ghrelin. Thirty participants per group (OSI and ORI) were required to detect a difference of 5% in the serial measurements of ghrelin with a power of 90% and alpha 0.05. Participant characteristics are presented as arithmetic means and standard deviations (SD). The results show the differences for sex adjusted for obesity resistance/susceptibility category (ORS) and differences for ORS adjusted for sex from regression analysis. A further adjustment %BF was conducted by including a term for %BF in the regression model. An interaction between sex and ORS group was considered but as it was not statistically significant it was not included in the final model. The fasting and AUC hormone variables were log transformed before analysis. The results are presented as medians (interquartile range). No adjustment was made for multiple testing. Statistical analysis was performed using STATA Version 12 (STATA Inc., College Station, TX).

## 3. Results

### 3.1. Participant Characteristics

The characteristics of study participants are shown in Table 1.

### 3.2. Hormone Analyses

Fasting, AUC, peak or nadir, and time to peak or nadir results for ghrelin, PYY (total), leptin, glucose, and insulin adjusted for ORS and sex with and without adjustment for %BF are presented in Table 2.

### 3.3. Ghrelin and PYY

No differences related to ORS or sex were observed in the analysis of ghrelin or PYY.

### 3.4. Leptin

Fasting leptin concentration and leptin AUC were significantly greater in females compared to males. The nadir for leptin was also higher in females compared with males. However, these differences disappeared when controlling for %BF. Fasting leptin concentration, leptin AUC, and nadir for leptin were all lower in ORI compared with OSI. Again, these differences disappeared when controlling for %BF. No differences were observed in the leptin time to nadir.

### 3.5. Glucose and Insulin

There were no significant differences for fasting, AUC, or time to peak for blood glucose for OSI versus ORI or males versus females. Peak blood glucose was significantly higher in males compared to females. Fasting insulin and insulin AUC were significantly lower in ORI compared with OSI. These differences disappeared when controlling for %BF.

### 3.6. Subjective Ratings of Hunger and Satiety

Fasting, AUC, peak or nadir, and time to peak or nadir results for “hunger,” “desire to eat,” “fullness,” and “preoccupation with thoughts of food” adjusted for ORS and sex with and without adjustment for %BF are presented in Table 3.

The nadir for “hunger” was significantly lower for OSI compared with ORI. The fasting rating of “preoccupation with thoughts of food” was significantly higher in females compared with males. Differences were also observed in the time to nadir for the “preoccupation with thoughts of food” rating, with the nadir occurring significantly later in ORI versus OSI. These differences disappeared after controlling for %BF. There were no significant differences observed in fasting, AUC, peak, or time to peak for ratings of “fullness” or “desire to eat.”

### 3.7. Three-Factor Eating Questionnaire (TFEQ)

**Figure 1:**
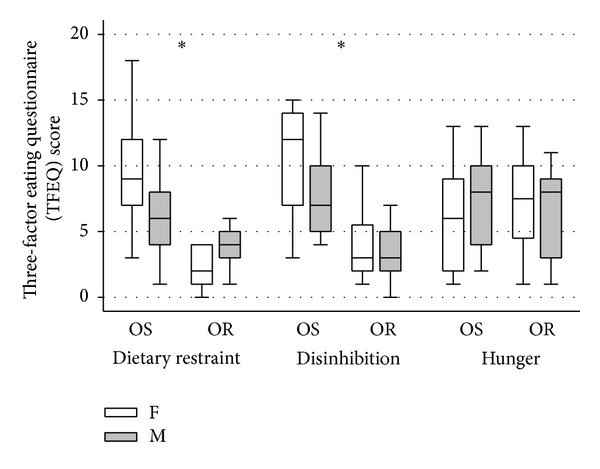
Three-factor eating questionnaire (TFEQ) scores for obesity resistant (OR) females (F) and males (M) versus obesity susceptible (OS) females (F) and males (M). Values are medians in 25th and 75th interquartile range. *= obesity resistant < obesity susceptible *P* < 0.001.

The TFEQ results are presented in Table 3 and Figure 1. Dietary restraint scores were significantly lower in ORI versus OSI (*P* < 0.001). Disinhibition scores were significantly lower in ORI versus OSI (*P* < 0.001), while no significant differences were observed in scores for hunger. These differences remained statistically significant after adjustment for %BF.

## 4. Discussion

Why some individuals remain lean with relative ease while others constantly struggle with their body weight, despite living in a similar environment, is an intriguing question. Although the majority of research in the obesity field has focused on the characteristics of obese individuals, an alternative approach is to compare the characteristics of those who are seemingly resistant to obesity (ORI) with those who appear highly susceptible (OSI). One reason for the difference in weight gain susceptibility may be due to physiological differences in appetite control. We studied the hormonal response to a standardized meal amongst ORI and OSI. Despite some differences in absolute values of these hormones, the patterns of change in response to a standard meal were remarkably similar between ORI and OSI.

As expected, ghrelin concentrations decreased in all groups upon feeding reaching a nadir between 30 and 60 min. Ghrelin, an orexigenic hormone, is acutely negatively regulated by the ingestion of meals and positively regulated by fluxes in overall energy balance [14]. Most previous studies have shown that total ghrelin is negatively correlated with %BF (obese individuals tend to have lower fasting ghrelin levels compared to lean controls) [17–19]. Based on these previous observations, one might have anticipated ORI (%BF: 23.4 in females, 15.4 in males) to have a higher fasting ghrelin compared to OSI (%BF: 41.9 in females, 27.6 in males). However, our study showed no differences in ghrelin concentration between ORI and OSI. Perhaps this is evidence that ORI and OSI respond differently, in terms of eating behaviour, to the same ghrelin concentration. That is, the OSI's higher level of dietary restraint and disinhibition may indicate that they are either more responsive to orexigenic signals and/or less responsive to anorectic factors.

Similar gherlin concentrations, despite different body composition and therefore energy stores, have also been observed in the study cohorts of two previous studies. Khoury et al. [28] compared gut hormone response to three standardized meals in a group with the metabolic syndrome and lean controls. Despite a 15% difference in mean body fat (33.5% versus 17.9%) there was no difference in active ghrelin. Mittelman et al. [19] showed that although fasting active ghrelin concentration was lower in obese, postprandial increases were similar in both lean and obese children.

The similarities in ghrelin concentration among the two groups may reflect two different mechanisms. Whereas the OSI ghrelin levels are due to increased energy stores, the ORI may have lower than predicted ghrelin levels due to a possible underlying protective mechanism which theoretically could protect against overeating and subsequent weight gain. Thus, no obvious difference between the two groups is apparent. Obesity resistance individuals may differ from other populations previously studied in that they largely struggle to gain weight rather than simply being lean. One previous study that investigated constitutionally thin (i.e., those who find it difficult to gain weight) also found lower ghrelin levels than expected given their low %BF [16]. As ghrelin is orexigenic this finding may indicate a possible mechanism that prevents these particularly lean individuals from overeating.

PYY is an anorexigenic hormone that has been associated with meal satiety and thus theoretically meal termination [15]. One may expect that those who remain lean with relative ease have a higher concentration of these hormones in response to feeding when compared to those who struggle to maintain a healthy body weight. In the majority of previous studies investigating overweight and/or obese compared to lean subjects, PYY levels were higher amongst lean individuals postprandially, suggesting a blunted response in overweight/obese individuals [16, 19, 20, 22, 24]. In contrast, we found no difference in PYY concentrations in response to a meal. This finding compliments the ghrelin results and may also indicate a differential response to the same PYY level in ORI versus OSI. Khoury et al. [28] also observed no differences in PYY responses and AUC between individuals with the metabolic syndrome and controls in response to a variety of meals.

Consistent with results from previous studies where higher leptin concentrations have been associated with increases in BMI [17, 29], the fasting and postprandial leptin concentrations in the present study were also higher in the group with greater BMI (OSI). This is in line with previous literature that has highlighted the concept of leptin resistance in overweight and obese individuals as a result of increased adipose tissue stores [30]. In our study OSI have markedly higher leptin concentrations than ORI but equivalent satiety scores. This suggests that OSI are not responding fully to the high concentrations of leptin.

In the face of similar hormone patterns it would have been of interest to observe how much our two groups would have eaten when presented with an* ad libitum* meal. Would they choose a similar meal size or would OSI have actually wanted to eat more? Future research should seek to investigate this as it represents a more realistic eating situation.

In addition to the potential differential response to the hormones in these two distinct groups of individuals there were some differences in perceived appetite ratings. Most notably, the ORI experienced smaller fluctuations in hunger ratings. In addition, the TFEQ indicates that ORI may respond differently to hunger in that they are less likely to engage in dietary restraint and disinhibition behaviours. This style of eating could be an artifact of the differential response to the hormones or may be in response to some psychological factors or learned behaviour. Overeating and lack of response to satiety cues may be a learned response that affects some to a greater extent than others. Physiological signals and behavioural cues both regulate appetite and energy intake. Which one predominates may depend on a number of factors including genetics, the environment, past experiences, parental influence, sensory stimulation, social situation, and psychological well-being.

There are some limitations that should be considered when interpreting the results of this study. Firstly, the cross-sectional design of the study does not allow us to draw casual inferences and the sample size was relatively small. Further, given that ghrelin plasma concentrations differ throughout the day in cyclic fashion in relation to meal taking and diurnal rhythms [14], it may be that the analyses simply did not capture ghrelin differences as values were only assessed over a 4 h period in the morning. In addition, any differences observed between males and females may be attributable to differences in the energy content of the standardized meal as this was based on sex rather than estimated energy requirements. This method for assigning energy content to the standardized meal could also potentially have resulted in the OSI eating less and the ORI eating more than they are used to. Furthermore, current dieting patterns may have influenced the results as individualswho were in a pattern of energy restriction may have responded differently compared to someone who was not actively trying to lose weight.

In conclusion, comparing people who remain lean despite living in an obesegenic environment (ORI) to those who struggle to maintain a healthy weight (OSI) has provided us with a novel approach to investigate the aetiology of obesity. Given the differential body weights observed in the two study groups in the present study, a similar ghrelin concentration was unexpected. This could indicate that OSI respond differently to the same ghrelin concentration. Conversely, the lower than expected fasting ghrelin levels observed in the ORI may provide a protective mechanism that enables these individuals to remain lean. This warrants further investigation. The higher levels of dietary restriction and disinhibition amongst OSI indicate that psychosocial factors are likely also important regulators of energy balance in this group.

## Figures and Tables

**Table 1:** Characteristics of obesity resistant individuals (ORI) and obesity susceptible individuals (OSI).

	ORI		OSI		*P* value*
	Females	Males	Females	Males	
*n *	16	17	15	13	
Age (years)	32.6 (7.6)	30.6 (7.7)	35.0 (7.7)	35.5 (9.1)	0.081
Weight (kg)	56.3 (5.3)	73.3 (10.7)	86.6 (15.2)	94.1 (11.0)	<0.001
Height (m)	1.65 (0.06)	1.81 (0.08)	1.66 (0.05)	1.79 (0.04)	0.400
WC (cm)	71.6 (6.0)	80.4 (4.7)	95.6 (10.8)	99.4 (11.7)	<0.001
BMI (kg/m^2^)	20.6 (1.8)	22.3 (2.9)	31.6 (6.2)	29.5 (3.3)	<0.001
LBM (kg)	40.1 (4.2)	58.5 (9.1)	45.8 (3.7)	63.7 (7.6)	0.002
Fat Mass (kg)	13.1 (2.9)	11.3 (4.6)	37.2 (14.2)	26.1 (8.1)	<0.001
% Body Fat	23.4 (4.8)	15.4 (5.6)	41.9 (9.9)	27.6 (7.1)	<0.001
TSH (*μ*IU·mL^−1^)	1.45 (0.96)	1.78 (0.97)	1.67 (0.77)	1.58 (0.82)	0.958

All values are means (standard deviation).

BMI: Body Mass Index, LBM: Lean Body Mass, TSH: Thyroid Stimulating Hormone (reference range = 0.3–5 *μ*IU·mL^−1^ for adults with no known thyroid dysfunction), WC: waist circumference.

*P value from regression analysis for ORS adjusted for sex.

**Table 2:** Hormone profiles in obesity resistant individuals (ORI) and obesity susceptible individuals (OSI) in response to a standardised meal.

	ORI						OSI						*P* value*	*P* value^†^	*P* value^‡^
	Females			Males			Females			Males					
	*n *	Median	Interquartile Range	*n *	Median	Interquartile Range	*n *	Median	Interquartile Range	*n *	Median	Interquartile Range			
Ghrelin															
Fasting (pg·mL^−1^)	14	70.7	39.2–129.5	17	55.2	38.6–137.3	12	69.3	59.5–105.5	12	55.0	35.6–65.1	0.319	0.600	0.662
AUC (mmol·min^−1^)	11	12596	7470–16987	16	11341	8747–21848	9	11232	9252–14968	9	9889	6136–10693	0.857	0.460	0.751
Nadir (pg·mL^−1^)	16	44.9	31.5–55.3	17	33.7	27.1–56.7	14	39.4	31.2–46.7	13	35.3	18.0–38.7	0.419	0.287	0.822
Time to nadir (min)	16	60	30–60	17	60	60-60	14	30	30–60	13	60	60-60	0.058	0.518	0.580
PYY															
Fasting (pg·mL^−1^)	14	47.9	40.1–53.7	17	55	47.8–65.3	14	47.1	37.5–67.3	12	56.4	49.4–62.0	0.161	0.889	0.761
AUC (mmol·min^−1^)	13	11700	10954–13230	16	12511	10524–13597	12	12779	10777–18752	9	12877	12436–13739	0.825	0.601	0.736
Peak (pg·mL^−1^)	16	72.6	68.2–82.0	17	77.8	66.7–87.5	15	75.4	55.0–112.4	13	75.7	68.7–87.5	0.981	0.877	0.916
Time to peak (min)	16	60	45–120	17	60	30–120	15	120	60–180	13	30	30-30	0.275	0.584	0.526
Leptin															
Fasting (pg·mL^−1^)	16	2426	1367–3626	17	693.4	440.9–1495.0	15	14367	4135–24941	12	2480	1424–8725	**<0.001**	**<0.001**	0.324
AUC (mmol·min^−1^)	16	425506	231672–582562	16	101878	74897–237294	14	1753714	502753–4671533	9	410572	268776–1746261	**<0.001**	**<0.001**	0.378
Nadir (pg·mL^−1^)	16	2003	1184–2986	17	531.7	339.7–1313.4	15	9015	2661–24450	13	2108	1198–6836	**0.003**	**0.001**	0.946
Time to nadir (min)	16	60	15–120	17	60	30–120	15	60	15–120	13	60	30–120	0.781	0.558	0.949
Glucose															
Fasting (mmol·L^−1^)	16	5.30	5.00–5.53	17	5.25	4.95–5.40	15	5.25	5.05–5.30	13	5.25	5.05–5.50	0.851	0.900	0.887
AUC (mmol·min^−1^)	16	1030	995–1063	17	1054	1013–1081	15	1062	970–1128	13	1048.0	992–1113	0.298	0.252	0.954
Peak (mmol·L^−1^)	16	6.9	6.70–7.55	17	7.3	7.00–8.00	15	7.2	6.7–7.8	13	7.5	7.10–7.60	**0.038**	0.540	0.759
Time to peak (min)	16	15	15–30	17	30	30-30	15	30	15–30	13	30	30-30	0.103	0.072	0.352
Insulin															
Fasting (pg·mL^−1^)	13	157	131.1–177.1	15	236.7	110.3–299.6	14	252.2	187.0–355.5	11	398	281.3–466.5	0.085	**0.004**	0.213
AUC (mmol·min^−1^)	12	105038	92640–143522	14	139943	108035–171234	12	175434	104685–217197	8	213250	131498–321104	0.057	**0.016**	0.802
Peak (pg·mL^−1^)	16	1345.1	965.9–1867.8	17	1677.5	1250.4–2298.3	15	1467.9	1098.9–2294.6	13	1849	1453–2475	0.145	0.196	0.632
Time to peak (min)	16	30	30-30	17	30	30-30	15	30	30-30	13	30	30-30	0.466	0.379	0.716

%BF: percent body fat, AUC: area under the curve, ORS: obesity resistance/susceptibility category.

Fasting and AUC data have been log transformed, *P value for sex adjusted for ORS, ^†^ *P* value for ORS adjusted for sex, ^‡^ *P* value for ORS adjusted for sex and %BF.

**Table 3:** Appetite ratings for obesity resistant individuals (ORI) and obesity susceptible individuals (OSI) in response to a standardised meal.

	ORI						OSI						*P* value*	*P* value^†^	*P* value^‡^
	Females			Males			Females			Males					
	*n *	Median	Interquartile Range	*n *	Median	Interquartile Range	*n *	Median	Interquartile Range	*n *	Median	Interquartile Range			
Hunger															
Fasting (mm)	16	73	55–86	17	67	49–84	15	65	51−77	13	60	40−70	0.515	0.074	0.341
AUC (mm·min^−1^)	16	9049	4819–10084	17	6413	4200–10358	15	4275	2798−6780	13	6158	5528−9443	0.506	0.056	0.785
Nadir (mm)	16	22	2–30	17	19	10–36	15	1	0−4	13	9	1−27	0.170	**0.017**	0.414
Time to Nadir (min)	16	15	15–30	17	30	15–60	15	15	15−30	13	15	15−30	0.656	0.305	0.252
Fullness															
Fasting (mm)	15	13	5–20	17	16	6–28	15	14	3-41	13	17	1−28	0.871	0.918	0.826
AUC (mm·min^−1^)	15	7095	6158–8670	17	6720	4800–10125	15	10185	6600−11295	13	7088	5595−7598	0.053	0.451	0.470
Peak (mm)	16	76	64–95	17	71	49–77	15	90	74−96	13	63	51−77	0.817	0.482	0.754
Time to peak (min)	16	15	15–30	17	15	15–30	15	15	15−30	13	30	15−30	0.380	0.119	0.860
Preoccupation with Food															
Fasting (mm)	16	63	49–85	17	34	28–63	15	57	30−70	13	41	15−68	**0.015**	0.448	0.987
AUC (mm·min^−1^)	16	6008	3173–8948	17	4628	2805–10830	15	2813	1875−7155	13	6660	2378−8663	0.496	0.294	0.455
Nadir (mm)	16	15	4–31	17	10	4–27	15	1	0−8	13	15	1−25	0.309	0.193	0.871
Time to Nadir (min)	15	15	15–30	17	30	15–30	15	15	15−30	13	15	15−60	0.092	**0.040**	0.888
Desire to Eat															
Fasting (mm)	16	76	58–85	17	64	45–85	15	69	49−79	13	61	35−72	0.336	0.199	0.243
AUC (mm·min^−1^)	16	8456	4943–10609	17	6473	4118–10913	15	3900	2753−6938	13	6705	5723−9788	0.389	0.148	0.427
Nadir (mm)	16	25	2–31	17	18	10–31	15	2	0−5	13	15	1−31	0.134	0.059	0.745
Time to Nadir (min)	16	15	15–30	17	15	15–30	15	15	15−30	13	30	15−60	0.770	0.923	0.940
TFEQ															
Dietary Restraint	16	4	0–12	17	4	3–5	15	9	3−18	13	6	4−8	0.337	**<0.001**	**<0.001**
Disinhibition	16	3	1–10	17	3	2–5	15	12	3−15	13	7	5−10	0.255	**<0.001**	**0.005**
Hunger	16	8	1–13	17	8	3–9	15	6	1−13	13	8	4−10	0.791	0.749	0.665

%BF: percent body fat, AUC: area under the curve, ORS: obesity resistance/susceptibility category, TFEQ: three factor eating questionnaire.

*P value for sex adjusted for ORS, ^†^ *P* value for ORS adjusted for sex, ^‡^ *P* value for ORS adjusted for sex and %BF.

## References

[1] World Health Organisation (2000). Obesity: preventing and managing the global epidemic. *A report of a WHO consultation*.

[2] Bajekal M, Borehan R, Erens B (1999). *Health Survey for England 1998*.

[3] Anderson JW, Konz EC, Frederich RC, Wood CL (2001). Long-term weight-loss maintenance: a meta-analysis of US studies. *American Journal of Clinical Nutrition*.

[4] Brown T, Kelly S, Summerbell C (2007). Prevention of obesity: a review of interventions. *Obesity Reviews*.

[5] Grodstein F, Levine R, Troy L, Spencer T, Colditz GA, Stampfer MJ (1996). Three-year follow-up of participants in a commercial weight loss program: can you keep it off?. *Archives of Internal Medicine*.

[6] Tsai AG, Wadden TA (2005). Systematic review: an evaluation of major commercial weight loss programs in the United States. *Annals of Internal Medicine*.

[7] Wang Y, Beydoun MA, Liang L, Caballero B, Kumanyika SK (2008). Will all Americans become overweight or obese? Estimating the progression and cost of the US obesity epidemic. *Obesity*.

[8] Flegal KM (1996). Trends in body weight and overweight in the U.S. population. *Nutrition Reviews*.

[9] Flegal KM, Troiano RP (2000). Changes in the distribution of body mass index of adults and children in the US population. *International Journal of Obesity*.

[10] Lewis CE, Smith DE, Wallace DD, Dale Williams O, Bild DE, Jacobs DR (1997). Seven-year trends in body weight and associations with lifestyle and behavioral characteristics in Black and White young adults: the CARDIA study. *American Journal of Public Health*.

[11] Lewis CE, Jacobs DR, McCreath H (2000). Weight gain continues in the 1990s 10-year trends in weight and overweight from the CARDIA study. *American Journal of Epidemiology*.

[12] Midthjell K, Krüger Ø, Holmen J (1999). Rapid changes in the prevalence obesity and known diabetes in an adult Norwegian population: the Nord-Trondelag Health Surveys: 1984–1986 and 1995–1997. *Diabetes Care*.

[13] Shah M, Hannan PJ, Jeffery RW (1991). Secular trend in body mass index in the adult population of three communities from the upper mid-western part of the USA: the Minnesota Heart Health Program. *International Journal of Obesity*.

[14] Cummings DE, Purnell JQ, Frayo RS, Schmidova K, Wisse BE, Weigle DS (2001). A preprandial rise in plasma ghrelin levels suggests a role in meal initiation in humans. *Diabetes*.

[15] Karra E, Chandarana K, Batterham RL (2009). The role of peptide YY in appetite regulation and obesity. *Journal of Physiology*.

[16] Germain N, Galusca B, Le Roux CW (2007). Constitutional thinness and lean anorexia nervosa display opposite concentrations of peptide YY, glucagon-like peptide 1, ghrelin, and leptin. *American Journal of Clinical Nutrition*.

[17] Beasley JM, Ange BA, Anderson CAM, Miller ER, Holbrook JT, Appel LJ (2009). Characteristics associated with fasting appetite hormones (obestatin, Ghrelin, and Leptin). *Obesity*.

[18] Le Roux CW, Patterson M, Vincent RP, Hunt C, Ghatei MA, Bloom SR (2005). Postprandial plasma ghrelin is suppressed proportional to meal calorie content in normal-weight but not obese subjects. *Journal of Clinical Endocrinology and Metabolism*.

[19] Mittelman SD, Klier K, Braun S, Azen C, Geffner ME, Buchanan TA (2010). Obese adolescents show impaired meal responses of the appetite-regulating hormones ghrelin and PYY. *Obesity*.

[20] Lomenick JP, Clasey JL, Anderson JW (2008). Meal-related changes in ghrelin, peptide YY, and appetite in normal weight and overweight children. *Obesity*.

[21] Geliebter A, Hashim SA, Gluck ME (2008). Appetite-related gut peptides, ghrelin, PYY, and GLP-1 in obese women with and without binge eating disorder (BED). *Physiology and Behavior*.

[22] Pfluger PT, Kampe J, Castaneda TR (2007). Effect of human body weight changes on circulating levels of peptide YY and peptide YY3-36. *Journal of Clinical Endocrinology and Metabolism*.

[23] Verdich C, Toubro S, Buemann B, Lysgård Madsen J, Juul Holst J, Astrup A (2001). The role of postprandial releases of insulin and incretin hormones in meal-induced satiety—effect of obesity and weight reduction. *International Journal of Obesity*.

[24] Stock S, Leichner P, Wong ACK (2005). Ghrelin, peptide YY, glucose-dependent insulinotropic polypeptide, and hunger responses to a mixed meal in anorexic, obese, and control female adolescents. *Journal of Clinical Endocrinology and Metabolism*.

[25] Blundell J, de Graaf C, Hulshof T (2010). Appetite control: methodological aspects of the evaluation of foods. *Obesity Reviews*.

[26] Stubbs RJ, Hughes DA, Johnstone AM (2000). The use of visual analogue scales to assess motivation to eat in human subjects: a review of their reliability and validity with an evaluation of new hand-held computerized systems for temporal tracking of appetite ratings. *British Journal of Nutrition*.

[27] Stunkard AJ, Messick S (1985). The three-factor eating questionnaire to measure dietary restraint, disinhibition and hunger. *Journal of Psychosomatic Research*.

[28] Khoury DE, Hwalla N, Frochot V, Lacorte J-M, Chabert M, Kalopissis AD (2010). Postprandial metabolic and hormonal responses of obese dyslipidemic subjects with metabolic syndrome to test meals, rich in carbohydrate, fat or protein. *Atherosclerosis*.

[29] Considine RV, Sinha MK, Heiman ML (1996). Serum immunoreactive-leptin concentrations in normal-weight and obese humans. *New England Journal of Medicine*.

[30] Caro JF, Sinha MK, Kolaczynski JW, Zhang PL, Considine RV (1996). Leptin: the tale of an obesity gene. *Diabetes*.

